# Regulation of Fruit Growth in a Peach Slow Ripening Phenotype

**DOI:** 10.3390/genes12040482

**Published:** 2021-03-26

**Authors:** Silvia Farinati, Cristian Forestan, Monica Canton, Giulio Galla, Claudio Bonghi, Serena Varotto

**Affiliations:** 1 Department of Agronomy, Food, Natural Resources, Animals and Environment (DAFNAE), University of Padova, Agripolis-Viale dell’Università 16, 35020 Legnaro, Italy; silvia.farinati@ipsp.cnr.it (S.F.); cristian.forestan@unibo.it (C.F.); monica.canton@phd.unipd.it (M.C.); serena.varotto@unipd.it (S.V.); 2 Institute for Sustainable Plant Protection (IPSP)—CNR, Agripolis-Viale dell’Università 16, 35020 Legnaro, Italy; 3 Dipartimento di Scienze e Tecnologie Agro-Alimentari (DISTAL), University of Bologna, Viale Fanin 44, 40127 Bologna, Italy; 4 Department of Biodiversity & Molecular Ecology, Research and Innovation Centre, Fondazione Edmund Mach, Via E. Mach 1, 38010 San Michele all’Adige, Italy; giulio.galla@fmach.it

**Keywords:** cell division, chromatin states, drupe, endoreduplication, fruit size, mesocarp gene identity, *Prunus*

## Abstract

Consumers’ choices are mainly based on fruit external characteristics such as the final size, weight, and shape. The majority of edible fruit are by tree fruit species, among which peach is the genomic and genetic reference for Prunus. In this research, we used a peach with a slow ripening (SR) phenotype, identified in the Fantasia (FAN) nectarine, associated with misregulation of genes involved in mesocarp identity and showing a reduction of final fruit size. By investigating the ploidy level, we observed a progressive increase in endoreduplication in mesocarp, which occurred in the late phases of FAN fruit development, but not in SR fruit. During fruit growth, we also detected that genes involved in endoreduplication were differentially modulated in FAN compared to SR. The differential transcriptional outputs were consistent with different chromatin states at loci of endoreduplication genes. The impaired expression of genes controlling cell cycle and endocycle as well as those claimed to play a role in fruit tissue identity result in the small final size of SR fruit.

## 1. Introduction

External characteristics of fruit such as the final size, weight, and shape are the key attributes used by consumers to identify and select preferred cultivars. Recently, it has been suggested that the manipulation of these attributes is a largely unused opportunity to create new and differentiated products [1].

The majority of edible fruit are species belonging to the Rosaceae family, for which studies evaluating fruit growth in the different stages showed that the final weight and size of the ripe fruit mainly depend on the number and size of mesocarp cells and intercellular spaces [2,3,4]. In particular, for peaches it has been reported that the final size is more related to the number of cells than to the enlargement process when large and small fruited-cultivars were compared [5]. In peach fruit, cell division is related to a fast growth rate, characterizing the first development phase (named S1). S1 duration varies among cultivars ranging from two [6] to five weeks [4] after full bloom. After the cell division cessation, the number of cells remains almost constant and the subsequent fruit growth is due to cell expansion [4]. Cell enlargement requires an intense cell wall remodeling, clearly visible four weeks AFB [6]. In agreement with this cytological observation, a decrease in cellulose deposition, paralleled by an increase in endoglucaneses activity [7], and a high activity of enzymes involved in cell wall strengthening, flexibility, and porosity such as xyloglucan endotransglycosylase and leucine-rich repeat family proteins/extensins, are observed during S1 [8]. The activity of these remodeling cell wall enzymes is almost zero during the following growth phase, called S2, in which growth is dramatically slowed and the endocarp is lignifying. The resumption of growth, occurring in the S3 stage after pit hardening, is sustained by cell enlargement, although at a lower rate in comparison to S1 [9]. Both at S1 and S3 stage, cell enlargement has been strictly related to auxin-induced growth processes [10,11]. Fruit cell expansion has also been related to cell ploidy in several species [12,13], by suggesting a role of endoreduplication in the control of fruit growth [14]. Differently from the majority of fleshy fruit for which the developmental cycle lasts for a long period (over 14 weeks), in peach fruit, together with apricot and plum, endoreduplication occurs [15]. However, in peach fruit the role of endoreduplication and its regulation is still poorly investigated. To fill this gap, the study of a clone from an open-pollinated population of Fantasia (FAN) nectarine (named BO80.004.106) showing a slow ripening (SR) phenotype has been [16,17].

SR trait is considered monogenic (Sr/sr) and when the recessive sr allele is in homozygosis, it prevents the fruit from undergoing normal ripening [18,19,20]. Recently, the SR locus has been mapped on linkage group 4 (G4) of peach genome and tightly linked molecular markers for its selection have been developed [21,22,23]. These markers are diagnostic of a large deletion of 26.6 kb containing the sequence of a NAC (Prupe.4G186800) transcription factor and this deletion is thought to cause the SR mutant phenotype. In addition to the slow repining phenotype, the SR population named SR-BO80.004.106 (from here on called SR), is also characterized by a small final fruit size, although the number of cells is higher in comparison to FAN fruit [24]. In addition, its phenotype is associated to an altered transcriptional regulation of genes involved in the mesocarp identity [24]. Taking into account these observations, SR can help to shed light on the molecular control of fruit growth.

Before the description of SR, the study of peach fruit size was restricted to the combination of RNA-Seq and QTL mapping. These approaches have been successfully used to determine critical genes for anthocyanin biosynthesis, stone adhesion, fleshy melting, and fruit swelling [25,26]. Fruit swelling has been related to fruit growth by linking nine highly transcribed genes, located within the QTLs for peach fruit size [27,28,29,30], to the swelling process [25]. In particular, genes associated with cellular cycle and cell wall turnover were identified. These genes are responsible in mediating cell number, and cell expansion [31]. In addition, a transcriptional model in which the abovementioned genes were linked to members of MADS-box, NO APICAL MERISTEM (NAC), TCP, basic leucine zipper (b-ZIP), and SUPERMAN transcription factor (TF) families, already associated with cell differentiation and cell division, was developed [32,33,34,35,36]. However, the transactivation of genes also requires promoters located to “open” chromatin domains. In addition, active chromatin is marked by a specific combination of modified core histone proteins (H3K27ac, H3K4me1, and H3K4me3) and by the presence of different histone variants [37]. Conversely, transcriptionally repressed genes are often located within “closed” chromatin domains, marked by specific histone modifications (e.g., H3K27me3 and H3K9me2) [38]. Studies on chromatin dynamics, such as changes in open chromatin states at target genes during fruit growth, could provide deeper insights into the molecular mechanisms of fruit size and patterning.

Here, we integrated different analyses able to characterize, in mesocarp cells, (1) the duration and rate of cell division and endoreduplication process (flow cytometry), (2) the expression of genes involved in cell division, endoreduplication, tissue identity, and markers of developmental phases, and (3) chromatin marks at target genes involved in the regulatory network of drupe growth. The final goal is to understand the involvement of the transcription regulation of target genes participating in mesocarp cells growth dynamics associated with fruit final size.

## 2. Materials and Methods

### 2.1. Plant Material, Samples, and Cytological Analysis

Ten-year-old *Prunus persica* (L. Batsch) trees of cv. FAN and the SR genotype (BO80.004.106, provided by Daniele Bassi, University of Milan), grown at the experimental farm ‘L. Toniolo’ of the University of Padova, Italy (GPS coordinates: 45°20′48.9″ N 11°57′00.3″ E) following the standard horticultural practices, were used. For each genotype, the fruit cross diameter of 100 fruits distributed on five trees was monitored on average weekly throughout development over the 2016 growth season, starting measurements 41 days after full bloom (DAFB), up to 132 DAFB to monitor the fruit growth. A total of 13 time points were obtained, i.e., at 41, 48, 55, 62, 69, 83, 90, 97, 104, 111, 118, 125, and 132 DAFB. On each sampling date, at least 15 fruits were always harvest from the same five trees (for each cultivar), and then the fruits were split in 3 biological replicates, each composed of 5 fruits. A mesocarp portion was collected taking from each fruit the whole radial section, or rather a portion of tissue characterized by a homogeneous cellular type, from the epicarp (sub-epidermal portion of mesocarp) to the first cell layer of endocarp tissue. After homogenization, each sample was stored at −80 °C for the following analyses.

For scanning electron microscopy (SEM), portions of mesocarp were fixed in 3% gluteraldehyde in 0.1 M cacodylate buffer, pH 6.9, for 16 h at 48 °C and post-fixed in 1% OsO4 in the same buffer for 1 h at 48 °C. Samples were dehydrated in an ethanol series to 100% ethanol, critical point dried, sputter coated with gold, and viewed with a Cambridge scanning electron microscope at an accelerating voltage of 150 kV. SEM images were analyzed, as reported by Souza et al. [39], to determine cell density and cell area.

### 2.2. Ploidy Analysis

For this analysis, the C-values were measured in a sample of mesocarp tissue derived from a pooling of 5 fruits for each genotype and for each time point for five replicates. Nuclei were isolated from approximately 100 mg of frozen mesocarp by gentle chopping with a razor blade in 0.4 mL of CyStain^®^ PI Absolute P nuclei extraction buffer (Sysmex Partec GmbH, Gorlitz, Germany) supplemented with 1% *w*/*v* PVP. Nuclei suspensions were filtered by using 30 μm CellTrics^®^ (Sysmex Partec GmbH, Gorlitz, Germany). Following the filtration step, 1.6 mL staining buffer was added to each sample and tubes were stored in the dark on ice for 1 h before measurement. The fluorescence intensity of DAPI-stained nuclei was determined using the flow cytometer CyFlow^®^ Cube Ploidy Analyzer (Sysmex Partec GmbH, Gorlitz, Germany) equipped with an UV-Light Emitting Diode (l = 355–375 nm). Data were plotted on a logarithmic scale and calibration of C values was made with nuclei suspensions prepared from young leaves. On average, 24,000 nuclei were assessed in each run. Ploidy histograms were quantitatively analyzed with the FCS Express 5 Flow software (Sysmex Partec GmbH, Gorlitz, Germany), after manual adjustment to exclude noise. For each ploidy level, significant differences in the number of nuclei were assessed by Student *t* tests.

### 2.3. RNA Isolation and qPCR Analysis

For expression analyses, at each time point for both genotype, total RNA extraction and cDNA synthesis were performed as reported in Botton et al. [24] 2016. qPCR was performed in triplicate on three biological replicates with a StepOnePlusTM Instrument (ThermoFisher), as described by Ziliotto et al. [40] and Eccher et al. [41].

The Phytozome database (http://phytozome.jgi.doe.gov, accessed on 30 January 2018) was used as a support for the download of *P. persica* transcript sequences and the primer design. The nucleotide sequences of the primers for both the target and reference genes are reported in Table S1.

Data were acquired, elaborated, and exported with the StepOne Software v2.3 (ThermoFisher, Waltham, MA, USA), whereas all final calculations were performed with the automated Excel spreadsheet Q-Gene designed by Simon [42], using the modifications of the delta Ct method suggested by Pfaffl [43].

Gene expression values were normalized to three housekeeping genes: TUB (Prupe.1G364800; [44], UBQ10 (Prupe.4G204900), and PpN1 (Prupe.8G137600; [45]), chosen because of their stability of expression as checked by means of the BestKeeper spreadsheet v1.0 [46]. Expression levels were then reported as arbitrary units (A.U.) of Mean Normalized Expression, calculated using the equation 2 of the Q-Gene spreadsheet.

### 2.4. Chromatin Extraction and ChIP Target Analysis

For chromatin extraction/purification and for the subsequent Chromatin Immunoprecipitation ChIP analyses, the samples considered were the fruit materials derived from collection in time points 83, 104, and 111 DAFB for both genotypes. By considering both the technical limits of the procedure and the cellular uniformity of the mesocarp tissue, the chromatin extraction procedure was performed after pooling of three biological replicates (with 5 fruits each). On the resulting biological sample (derived from a total of 15 fruits), three independent immunoprecipitation reactions were performed.

For each available sample, the frozen mesocarp tissue was finely powdered with liquid nitrogen and the chromatin was extracted by transferring the fine powder into a 50 mL tube and fixing with 1% formaldehyde in cold Nuclear Isolation Buffer (NIB, 10 mM HEPES pH 7.6, 1 M sucrose, 5 mM KCl, 5 mM MgCl_2_, 5 mM EDTA, 0.6% Triton X-100, 0.5 mM PMSF, 5% PVP) at a ratio 1.5 g of tissue/25 mL of NIB. Fixation was done for 15′ at RT and then blocked for 5′ with 3.4 mL of 1M Glycine. Subsequently, the lysate was filtered through a single layer of Miracloth (Millipore) into a new tube and centrifuged at 5000× *g* for 15′ at 4 °C. The white pellet was washed twice with 1–2 mL of cold NIB without the addition of both Triton X-100 and PMSF. The chromatin pellet was then suspended in 5 mL of 15% Percoll solution (15% Percoll, 10 mM HEPES pH 7.6, 1 M sucrose, 5 mM KCl, 5 mM MgCl_2_, 5 mM EDTA, 5% PVP). The suspension was centrifuged at 5000× *g* for 15′ at 4 °C and finally the pellet was suspended in 500µl of Lysis Buffer (50 mM HEPES pH 7.6, 150 mM NaCl, 1 mM EDTA, 1% Triton X-100, 0.1% deoxycholate, 0.1% SDS, 10mM Na-butyrate, and protease inhibitor cocktail (Sigma). The extracted chromatin was sonicated to 200–300 bp fragments and centrifuged at 16,000× *g* for 10′ at 4 °C. A fraction of the supernatant was saved and after its reverse cross-linking with 0.2 M NaCl for 16 h at 65 °C, used for quantification and as input in the following PCR evaluation. For immunoprecipitation, 10 µg of chromatin was used for each antibody precipitation reaction. The histone-DNA complexes were immunoprecipitated overnight at 4 °C adding the appropriate antibody: α-H3K9ac (Millipore, Cat. 07-352), α-H3K4me3 (Active Motif, Cat. 39159), and α- H3K27me3 (Millipore, Cat. 07-449). The antibody incubation and pre-clarification step for 3 h at 4 °C were performed using Dynabeads protein G (Invitrogen). A chromatin aliquot processed like other samples but without the addition of any antibody (No Ab sample) was used as background control. The beads linked to histone-DNA immunoprecipitated complexes were then washed sequentially, once with Low Salt Buffer (20 mM Tris-HCl pH 8.0, 150 mM NaCl, 1% Triton X-100, 2 mM EDTA, 0.1% SDS), High Salt Buffer (20 mM Tris-HCl pH 8.0, 500 mM NaCl, 1% Triton X-100, 2 mM EDTA, 0.1% SDS), LNDET (20 mM Tris-HCl pH 8.0, 250 mM LiCl, 1% Nonidet P-40, 1% deoxycholate), and twice with TE (Tris-EDTA) Buffer. The chromatin was eluted with 0.1M NaHCO3 and 1% SDS and the cross-linking was reversed with 0.2 M NaCl for 16 h at 65 °C. The DNA samples were precipitated with Glycogen (Sigma) and 2 volumes of absolute ethanol for 2h at −20 °C. After pellet collection and its washing with 70% ethanol, DNA was resuspended in TE Buffer and treated with RNAse I for 30′ at 37 °C and with proteinase K for 1 h at 42 °C, extracted once with phenol-chloroform and with QIAquick PCR purification kit (QIAGEN). One microliter of this ChIPed DNA and an appropriate dilution of input were used for the following qPCR analyses. For each target gene investigated, at least two pairs of primers were considered for PCR reactions, and the sequences of best working primers (in terms of dimer formation and specificity), with relative coordinates on locus, are reported in Table S1. qPCR data analyses were performed as reported in Rossi et al. [47] and significant differences in the level of each analyzed histone mark were assessed by Student *t* tests.

## 3. Results

### 3.1. Fruit Growth Pattern in FAN and SR

**Figure 1:**
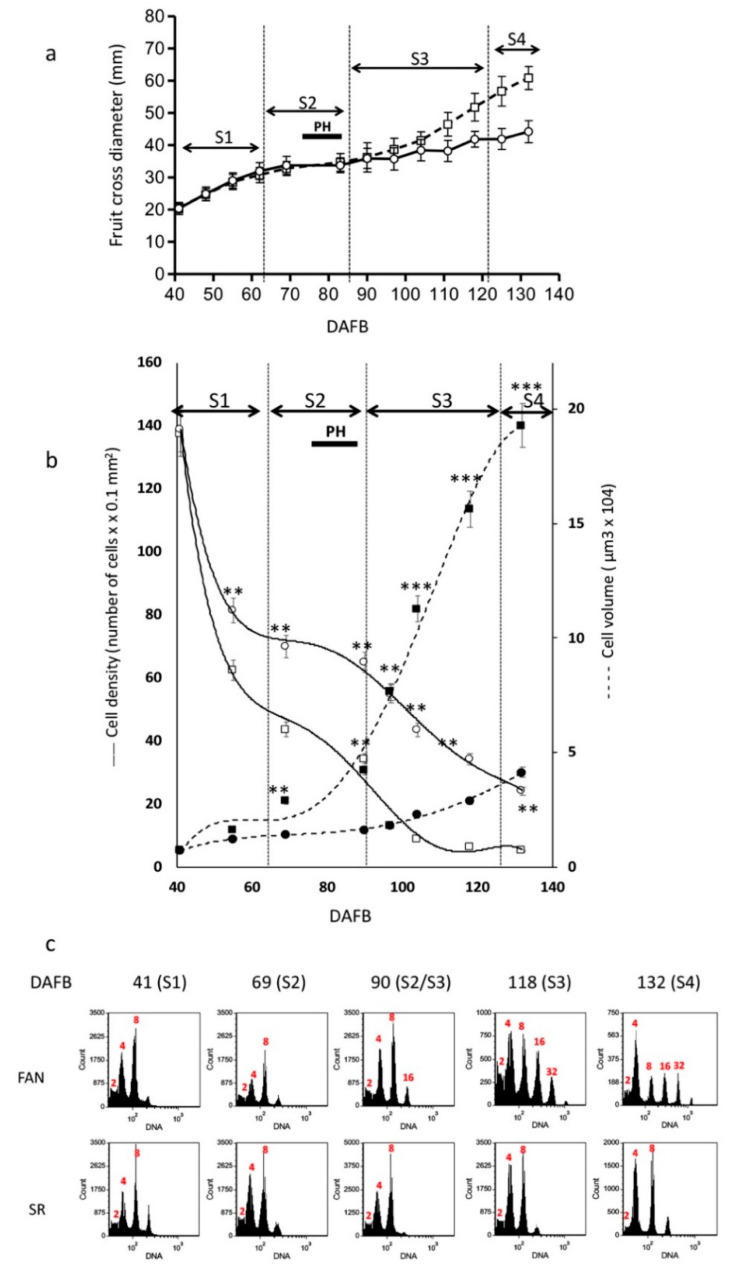
Fruit growth curve of cv FANTASIA (FAN) and slow ripening (SR) genotype. Cell density and volume, and cell cycle assessment by flow cytometry in mesocarp cells of FAN and SR were measured in samples collected in 2016, during the growth period from 41 to 132 days after full bloom (DAFB). (**a**) Diameter kinetic of FAN and SR fruits, represented by squares and dashed line and circles and continuous line, respectively. Bars represent standard deviation (n = 100). (**b**) Developmental changes in number of cells per 0.1 mm^2^ and cell volume in FAN (square) and SR (circle) fruit. Standard errors are reported (*n* = 5 fruits). Asterisks indicate statistically significant changes: ** = *p* ≤ 0.01, *** = *p* ≤ 0.0015. (**c**) Flow cytometry (FCM) analyses performed at different stages during FAN and SR fruit development. Red number indicates DNA content/ploidy (2C, 4C, 8C, 16 C, and 32C). Developmental stages (S1, S2, S3, and S4) are indicated at the top of each chart, taking FAN as a reference. DAFB, days after full bloom. PH, pit hardening.

Fruit growth analysis was performed on cv FAN, assumed as reference for all our analyses, and SR genotypes. FAN and SR fruit size was assessed throughout the growth process in 2016, from 41 to 132 days after full bloom (DAFB), comprising a time period from the initial growth phase to the beginning of ripening time for cv FAN (Figure 1a).

FAN fruit developmental kinetic follows the typical double sigmoidal pattern, in which the four developmental stages S1, S2, S3, and S4 were identified as defined by Tonutti et al. [48]. S1 lasts up to 60 DAFB, and S2, in which the pit hardening (PH) occurs, is completed by the S2/S3 transition at 85 DAFB, whereas ripening (S4 stage) begins at 125 DAFB. For SR fruit, the developmental abnormalities in fruit growth kinetic reported in Botton et al. [18] were confirmed. In particular, SR fruit kinetic substantially overlaps that of FAN up to early S3 (90 DAFB). Later on, from 97 to 132 DAFB, the average growth rate (0.24 mm/day) of SR was lower than that observed in FAN (0.63 mm/day). This growth rate slowdown resulted in a smaller SR fruit size (−28% in comparison to FAN) at the onset of S4. As expected, in FAN fruit cell density rapidly decreases after the cessation of cell division, while cell volume rapidly increases with the resumption of growth in S3. In SR fruit, these two parameters were highly perturbed because cell density decreased slowly, and cell volume was almost constant with an increasing trend at S4 (Figure 1b).

### 3.2. Altered Levels of Ploidy Were Observed in SR Mesocarp

In SR fruit, the slow decrease of cell density and smaller cell volume in comparison to FAN fruit account for an alteration of cell growth processes (Figure 1a,b). To investigate the relationship between cell size, determined by cell division and endoreduplication, and fruit size, the DNA content of mesocarp cells was measured by flow cytometry in FAN and SR fruits at each time point of sample collection. At 41 DAFB, in S1 stage, the two genotypes displayed similar profiles of DNA content profiles with cell C values ranging mainly from 2 to 8C. In both genotypes, cells with a DNA content of 4C were the most represented, accounting for 53.2-56%, while about 31% of cells had a DNA content of 2C (Figure 1c and Figure S1). The high proportion of 4C cells pointed out that both cells were undergoing division (G2/M phase) and cells that had undergone one cycle of endoreduplication were present. At this stage, events of endoreduplication were indicated by a DNA content of 8C (8–13%), clearly representing cells that underwent two cycles of endoreduplication. At 69 DAFB, during S2 stage, the C value proportions observed were similar to those measured in S1 and no significant difference, on the basis of *t*-test (Table S2), was detected by comparing FAN vs. SR fruits (Figure 1c). It is worth noting that during the S2 phase, all growth processes occurring in mesocarp cells were strongly slowed down due to the diversion of energy resources towards the endocarp for its lignification (Figure 1a). At 90 DAFB, mesocarp cells collected from cv FAN with a DNA content of 8C, 16C, and 32C were progressively increasing reached a proportion of 22%, 8, and 0.5% respectively; while SR mesocarp cells retained the cytofluorimetric profile observed in S2. FAN mesocarp cells at S4 (132 DAFB) showed a further increase of 16C (13%) and 32C (2.4%) proportions, counterbalanced by a decrease in the abundance of the 4C population (19.8%), while the level 2C and 8C fractions remained stable. Remarkably, the cytofluorimetric profile observed in SR, at stage S4, was not different from that observed in early stages.

Our cytofluorimetric analysis indicated that in S1 and S2, the composition of cell populations, in terms of DNA content, was similar between the two genotypes, although the cell volume in FAN fruits was already significantly higher than that observed in SR (Figure 1b). Later on, the progressive increase of cell proportion undergoing 4–5 endocycles was occurring only in S3 and S4 FAN fruit, suggesting that endoreduplication may be associated to the higher volume registered in FAN fruit (Figure 1b).

### 3.3. Expression Level of Cell Cycle and Endoreduplication-Related Genes Is Altered in SR Mesocarp Tissue

**Figure 2:**
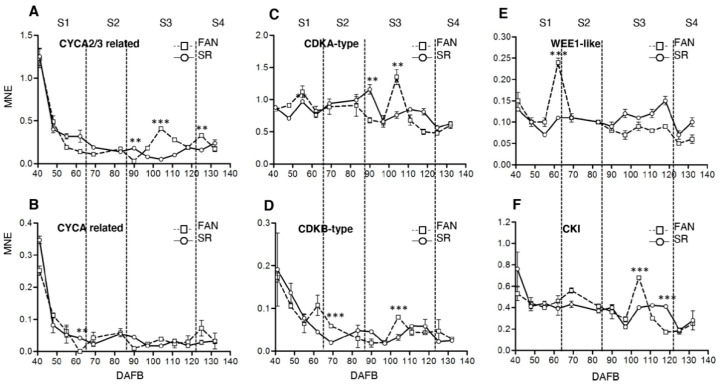
Expression analysis of selected target genes controlling cell cycle and endoreduplication process during the growth period of FANTASIA (FAN) and slow ripening (SR) genotype fruits. Real-time qPCR expression analysis of CYCA2/3 peach ortholog (Prupe.3G075200, Panel **A**), CYCA related (Prupe.1G428100, Panel **B**), CDKA-type (Prupe.2G084600, Panel **C**), CDKB-type (Prupe.6G299900, Panel **D**), WEE1 (Prupe.8G256300, Panel **E**), and CKI (Prupe.1G006300, Panel **F**) genes throughout fruit development in the mesocarp of cv FAN (square, dashed line) and SR (circle, continuous line) (for details and primers sequences see Methods). Developmental Scheme 1. S2, S3, and S4) are indicated at the top of each chart, taking FAN as a reference. Highly and extremely significant differences as determined by Student’s *t* test are indicated with two (*p* ≤ 0.01) and three (*p* ≤ 0.001) asterisks, respectively. MNE: Mean normalized expression, DAFB: Days after full bloom. Bars represent standard deviation (*n* = 3).

By considering the differences in ploidy level during fruit development in FAN and SR (Figure 1c), the expression analysis of some of these important marker genes related to cell cycle progression and endoreduplication processes (in/directly correlated to cell ploidy level) in Rosaceae fruits [49,50], was investigated through qPCR. In more detail, the transcript level of cyclins (CYCA2/3 related, Prupe.3G075200; CYCA-related, Prupe.1G428100, Prupe.1G430500), cyclin-dependent kinase (CDKA-type, Prupe.2G084600; CDKB-type Prupe.6G299900), WEE1-like protein (Prupe.8G256300), and CYCLIN-DEPENDENT KINASE INHIBITOR 1 (CKI, Prupe.1G006300) was measured in both genotypes during fruit development (Figure 2).

**Figure 3:**
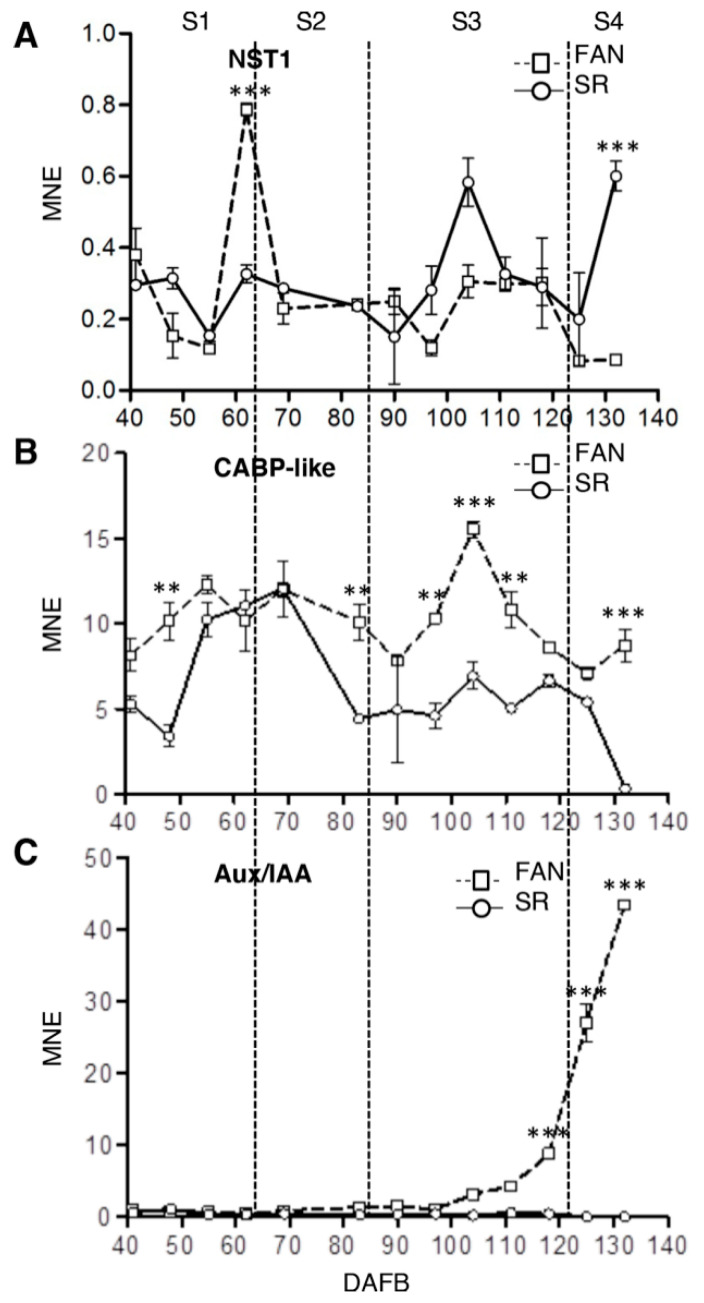
Expression analysis of selected target genes controlling fruit identity tissue and marking fruit development phases during the growth period of FANTASIA (FAN) and low ripening (SR) genotype fruits. Real-time qPCR expression analysis of peach ortholog of NST1(Prupe.5G131900, Panel **A**), CABP-like (Prupe.5G117500, Panel **B**), and Aux/IAA (Prupe.8G232200, Panel **C**) genes throughout fruit development in the mesocarp of cv. FAN (square, dashed line) and SR (circle, continuous line) (for details and primer sequences see Methods). Developmental stages (S1, S2, S3, and S4) are indicated at the top of each chart, taking FAN as a reference. Highly and extremely significant differences as determined by Student’s *t* test are indicated with two (*p* ≤ 0.01) and three (*p* ≤ 0.001) asterisks, respectively. MNE: Mean normalized expression, DAFB: Days after full bloom. Bars represent standard deviation (*n* = 3).

Cell cycle progression is controlled by the periodic activity of heterodimeric threonine/serine protein kinases composed of catalytic and regulatory subunits, a CDK and a cyclin, respectively. At 41 DAFB in FAN fruit, transcripts encoding for CYCA2/3-re, CYCA, and CDKB-type were more abundant than in other samples collected in S1 (Figure 2A,B,D). This result suggests that the majority of l cells of the 4C population were progressing in the cellular cycle. Later on, up to the end of S2, the transcript level of all genes dropped down with the exception of CDKA-type that remained at a steady state (Figure 2A–D). During S3m a transcript level increase was recorded for both CDKs (Figure 3C,D) and CYCA2/3 (Figure 2A). In SR, the transcript accumulation patterns of CYC and CDK overlapped those observed in FAN until the end of S2, while in S3, a delay of transcript upregulation (to a lesser extent) occurred (Figure 2A–D).

In FAN, the transcript level of the mitosis blocker WEE1-like protein significantly peaked (*p* ≤ 0.001) at the end of S1 (Figure 2E) suggesting that cells with a DNA content of 4C, the most represented up to the end of S2 (Figure 1c), were preferably addressed towards the endocycle. In the subsequent developmental phases, WEE1-like gene expression showed a decreasing trend. In SR, the transcriptional up-regulation of WEE1-like protein was lacking, and the expression remained at a steady-state level during the fruit development (Figure 2E). In FAN, a clear upregulation of CKI expression (*p* ≤ 0.001) was noted at S3 between 90-110 DAFB (Figure 2F). This expression trend overlapped that observed for CDKA-type and is very similar to that of CYCA2/3.

Taken together, these results indicate that the differences in gene transcripts level between the two genotypes are consistent during S3, in which the differences in the composition of cells in terms of DNA content are also significant (Figure 1c). However, it is worth noting that the transcript level increases of the mitosis blocker WEE1-like at the end of S1 in FAN fruit might be associated endoreduplication events occurring.

### 3.4. Altered Expression Level of Developmental Stage and Organ Specific Markers in SR Mesocarp Tissue

Previous observations and comparisons between FAN and SR fruit pointed out that mesocarp development was different starting from S2/S3 transition stage (Botton et al., 2016). This difference can be associated with either a ‘delay in phase transition starting from S3′ or to a ‘misregulation of tissue identity’ occurring in SR fruit. To verify these hypotheses and link them to the observed defects in endo-reduplication event in SR, the expression level of both developmental phases of mesocarp-specific genes [45] and an endocarp-specific marker gene [51] was measured by real-time PCR in both genotypes (Figure 3).

**Figure 4:**
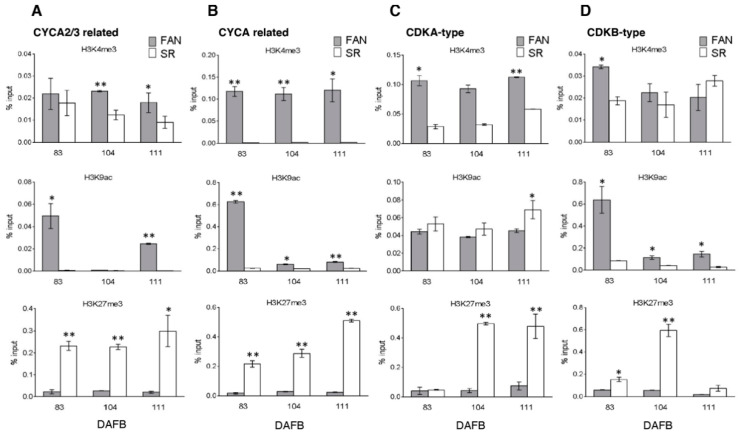
Chromatin mark analysis at target genes controlling cell cycle during the growth period of FANTASIA (FAN) and slow ripening (SR) genotype fruits. Histone modification analysis at CYCA2/3 related (Prupe.3G075200, (Panel **A**)), CYCA related (Prupe.1G428100, Panel **B**), CDKA-type (Prupe.2G084600, Panel **C**), CDKB-type (Prupe.6G299900, Panel **D**) loci by real-time PCR quantification of ChIPed DNA immunoprecipitated with α-H3K4me3, α-H3K9ac, and α-H3K27me3 antibodies on chromatin extracted from FAN (grey bars) and SR (white bars) mesocarp tissue at 83, 104, and 111 DAFB. Data are reported as percentage of chromatin input, normalized on background signal (measured by omitting antibody during ChIP procedure) and three PCR repetitions for each ChIP assay. Standard errors are reported. Asterisks indicate statistically significant changes: * = *p* ≤ 0.05, ** = *p* ≤ 0.01. DAFB: Days after full bloom.

The expression of NO SECONDARY WALL THICKENING (NST1, Prupe.5G131900), the endocarp marker gene, showed a peculiar trend in SR mainly during S1/S2 transition stage and in the middle of S3 phase. In fact, while in FAN fruit the transcript level of NST1 reached its maximum around 60 DAFB, and then it remained at lower constant level for the entire fruit growth, in SR NST1, the peak of the transcript was detected at 104 DAFB in S3 and then the transcript level increased again during S4 stage (Figure 4A). The expression of Prupe.5G117500, which encodes for a putative endoplasmic reticulum-located, histidine-rich, Ca2+ binding protein (CABP-like), had a maximum in FAN at S3 (104 DAFB), while expression levels were much lower in the previous and following stages. In SR, the accumulation of SP transcripts steadily increased during the early phases up to a maximum at S2, but the peak observed in FAN during S3 was not detected in SR (Figure 4B). S4 stage is clearly identified by the expression of Prupe.8G232200 coding for an Aux/IAA protein. In FAN, its expression began to increase from S3 stage (104 and 111 DAFB) to reach a maximum at the end of growth. Differently, in SR it was almost undetectable in all developmental phases (Figure 4C).

In conclusion, the expression profile both of genes involved in tissue identity and markers of developmental phase of fruit indicate that SR mesocarp looks like an endocarp and its development is almost blocked at S3 stage.

### 3.5. Differential Enrichments and Depletion of H3K4me3, H3K9ac, and H3K27me3 Modified Histones Were Detectable in Cell Cycle, Endoreduplication-Related and Tissue Marker Gene Loci

Both genes involved in cell cycle and endoreduplication together with genes that marked specific fruit developmental phases and tissues, showed different expression patterns in SR compared to FAN. In order to verify the chromatin state of these differentially expressed genes in the two genotypes, we investigated the distribution of different chromatin marks through Chromatin Immuno Precipitation (ChIP) at target gene loci, because a correlation is defined. between plant gene transcription and distinct histone modifications deposition. A region at gene 5′-end around the annotated TSS was investigated and considered as nucleotide region for the primers design used during ChIP investigations.

ChIP was performed using antibodies against H3K4me3 and H3K9ac modified histones for activating marks and H3K27me3 modified histone as repressing mark. The ChIPed DNA was then quantified by qPCR and the specific primer sequences used for each gene locus are reported in Table S1.

By considering cytofluorimetric and gene expression results, we focused our attention on the S3 phase of fruit development, which is marked by endoreduplication events and where morphological differences between the two genotypes are more evident. Hence, for both genotypes, the analysis was performed on fruit sampled at 83, 104, and 111 DAFB.

For the CYCA2/3 locus (Prupe.3G075200), different histone modification accumulation profiles were observed in the two genotypes, at the three considered time points. In particular, a persistent higher enrichment of H3K4me3 was observed in FAN vs. SR at 83, 104 and 111 DAFB, with a major difference at 104 DAFB. A significant difference in the enrichment of H3K9ac was also detected, with extremely low accumulation level in SR both at 83 (*p* ≤ 0.05) and 111 (*p* ≤ 0.01) in comparison to FAN. Enrichment levels were comparable in both genotypes only at 104 DAFB. On the contrary, in SR, a significant (*p* ≤ 0.01 at 83 and 104 DAFB) and permanent enrichment of H3K27me3 was detected in all three tested time points (Figure 4A).

A similar profile of chromatin mark distribution was observed for a CYCA3 coding gene (Prupe.1G428100). In comparison to FAN, a low level of H3K4me3 modified histone was detected in SR during fruit growth; a similar profile was also observed for H3K9ac, although an evident decreasing trend for this mark was detected in FAN after 83 DAFB. An opposite trend was observed for H3K27me3 modified histone, which was enriched in SR compared to FAN, with increasing modification levels in SR and constant lower levels in FAN, during fruit growth (Figure 4B).

CDKA-type (Prupe.2G084600) and CDKB-type (Prupe.6G299900) coding loci also showed different enrichment profiles in the two genotypes. At the CDKA-type locus (Prupe.2G084600), the two activating marks, H3K4me3 and H3K9ac, did not show modulations during fruit growth (from 83 to 111 DAFB) in either genotype. However, lower levels of H3K4me3 were measured in SR, whereas no significant difference (*p* ≤ 0.05) was observed between FAN and SR for H3K9ac levels. Conversely, an enrichment in H3K27me3 was observed in S3 phase in SR (104 and 111 vs. 83 DAFB) (Figure 4C).

At CDKB-type locus different chromatin profiles were observed: For H4K4me3 a slight difference was detected between FAN vs. SR at 83 DAFB, while similar enrichment levels were observed at 104 and 111 DAFB in both genotypes. A significant difference was instead observed for H3K9ac: A strong decrease of this mark level was observed in FAN after the S2/S3 transition phase (83 DAFB) and from this time point, a lower level was observed in all tested SR samples. Interestingly in SR, a peculiar clear (*p* ≤ 0.01) enrichment peak was detected for H3K27me3 at 104 DAFB (Figure 4D).

**Figure 5:**
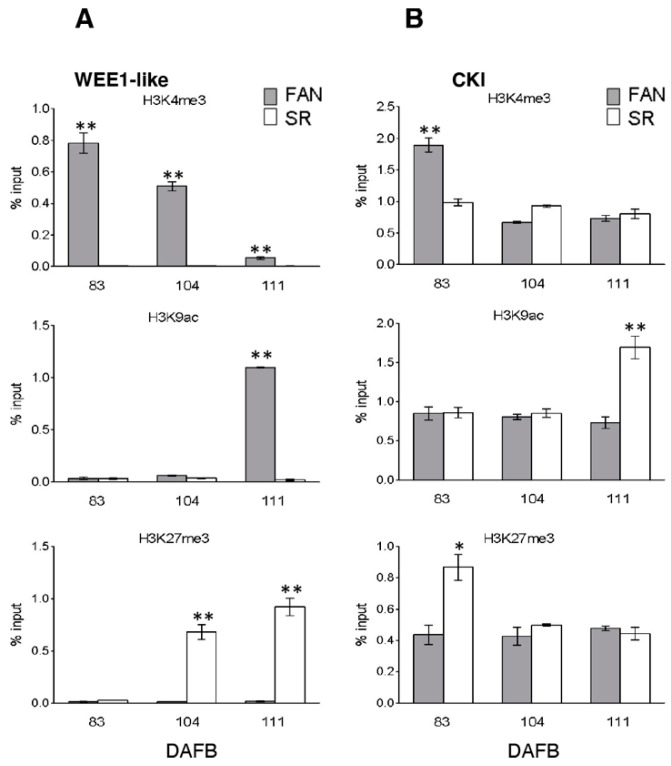
Chromatin mark analysis at target genes controlling endoreduplication during the growth period of FANTASIA (FAN) and slow ripening (SR) genotype fruits. Histone modification analysis at WEE1 (Prupe.8G256300, Panel **A**) and CKI (Prupe.1G006300, Panel **B**) loci by real-time PCR quantification of ChIPed DNA immunoprecipitated with α-H3K4me3, α-H3K9ac and α-H3K27me3 antibodies on chromatin extracted from FAN (grey bars) and SR (white bars) mesocarp tissue at 83, 104, and 111 DAFB. Data are reported as percentage of chromatin input, normalized on background signal (measured by omitting antibody during ChIP procedure), and three PCR repetitions for each ChIP assay. Standard errors are reported. Asterisks indicate statistically significant changes: * = *p* ≤ 0.05, ** = *p* ≤ 0.01. DAFB: Days after full bloom.

ChIP analysis was also performed for the two genes involved in the endoreduplication process, WEE1-like (Prupe.8G256300) and CKI (Prupe.1G006300) (Figure 5).

At WEE1-like gene, opposite profiles were observed in FAN compared to SR for all three tested modified histones: In SR, a complete depletion of H3K4me3 and extremely low levels for H3K9ac were observed in all three time points. A complementary profile was detectable for H3K27me3 that was particularly enriched at S3 phase (104 and 111 DAFB) in SR compared to FAN in which H3K27me3 levels were almost undetectable in each time point (Figure 5A).

At the CKI locus, a depletion of H3K4me3 mark level was observed in FAN during the course of fruit development, with an initial peak at 83 DAFB. For the same mark in SR, no difference was detectable in the three samples, with H3K4me3 levels comparable to FAN at 104 and 111 DAFB. In FAN and SR, H3K9ac showed comparable levels in fruit sampled at 83 and 104 DAFB, whereas an enrichment of H3K9ac was observed in SR fruit at 111 DAFB. Interestingly an enrichment of H3K27me3 in 83 DAFB sample was observed in SR (Figure 5B).

**Figure 6:**
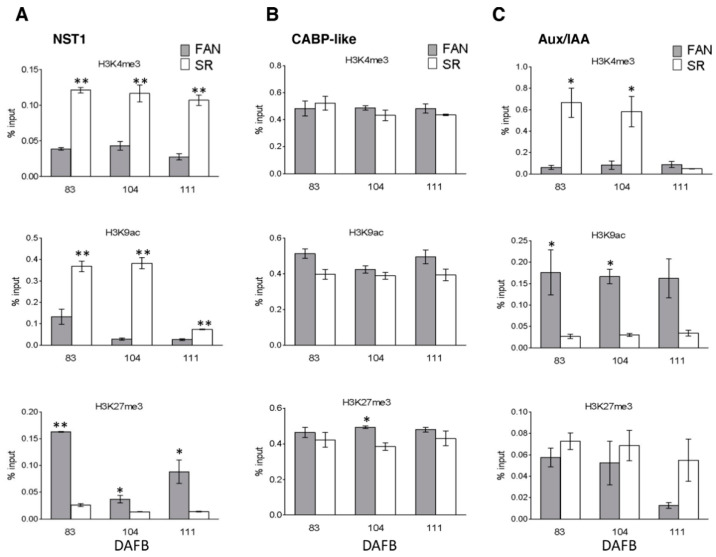
Chromatin mark analysis during the growth period of FANTASIA (FAN) and slow ripening (SR) genotype fruits, at target genes controlling fruit identity tissue and marking fruit development phases. Histone modification analysis at NST1 (Prupe.5G131900, Panel **A**), CABP-like (Prupe.5G117500, Panel **B**), and Aux/IAA (Prupe.8G232200, Panel **C**) loci by Real-time PCR quantification of ChIPed DNA immunoprecipitated with α-H3K4me3, α-H3K9ac and α-H3K27me3 antibodies on chromatin extracted from FAN (grey bars) and SR (white bars) mesocarp tissue at 83, 104, and 111 DAFB. Data are reported as percentage of chromatin input (normalized on background signal) measured by omitting antibody during ChIP procedure), and three PCR repetitions for each ChIP assay. Standard errors are reported. Asterisks indicate statistically significant changes: * = *p* ≤ 0.05, ** = *p* ≤ 0.01. DAFB: Days after full bloom.

At the NST1 locus (Prupe.5G131900), a persistent and significantly (*p* ≤ 0.01) higher level of H3K4me3 was observed in SR compared to FAN during the fruit development. In SR, a higher level of H3K9ac was also detected at 83 and 104 DAFB. Conversely, enrichment in H3K27me3 was detected in FAN compared to SR that showed a significantly lower level of H3K27me3 in all time points, in particular (*p* ≤ 0.01) at 83 DAFB (Figure 6A).

ChIP analysis did not show significant differences in the three marks investigated at CABP-like gene locus (Prupe.5G117500) (Figure 6B). At the Aux/IAA gene locus (Prupe.8G232200), H3K4me3 modified histone was enriched in SR at 83 and 111 DAFB. H3K9ac was highly enriched in FAN at the three time points considered. This gene locus was enriched in H3K27me3 both in FAN and SR at 83 and 104, whereas a depletion of H3K27me3 was detected in FAN at 111 (Figure 6C).

Taken together, these results highlight that modified histone enrichment and depletion at specific target loci are consistent with gene modulation at expression level in both FAN and SR.

## 4. Discussion

Fruit growth is a complex developmental process during the plant life cycle. Throughout the process, spatial and temporal events occur starting from ovary formation, fruit set, and fruit ripening: Each phase is characterized by specific gene expression profiles [52]. For these reasons, the implementation of both genetic and molecular studies is necessary to implement available information. Investigations on spontaneous mutants can be extremely useful for research purposes in pomology.

### 4.1. Endoreduplication Processes Slight Affect Peach Fruits Size

In our case, the use of the SR clone in which the fruit growth is altered allowed a better dissection of mechanisms governing a critical developmental phase from a biological and economical point of view. Preliminary genomic sequencing data pointed out that the deletion of 26.6 kb containing the sequence of a NAC (Prupe.4G186800) transcription factor [22] is not present in the genome of our SR clone [53] although the transcript of NAC gene is barely detectable in SR developing fruit (Figure S2). Therefore, in our clone, the sr/sr allelic combination at the SR locus might suppress NAC transcription through other structural rearrangements or regulatory mechanisms not involving genomic DNA changes. Botton et al. [24] proposed that the peach HEC3-like gene FLESHY, by having an important role in fruit tissue patterning and in early fruit development, might be involved in the SR phenotype determination. This hypothesis corroborates the idea that SR phenotype might not be controlled by a single ‘major gene’ but that a broad disruption of regulatory mechanisms of fruit developmental pattern might be associated with the mutation. The impaired expression of NAC gene could only partially explain the fruit ripening altered phenotype [20], while the causes of altered fruit growth kinetics, which determine a reduced fruit size, are still obscure. Our preliminary hypothesis was that a possible cause of SR fruit size reduction could be related to an alteration of cell ploidy profile in comparison to FAN. In FAN, a possible contribution of ploidy level in the control of cell (and consequently organ) size was supposedly following the karyoplasmic ratio theory. This theory states that cells tend to adjust their cytoplasmic volume to the nuclear DNA content, as reported for tomato fruit [54]. Cytofluorimetric analysis confirms that in peach fruit up to 4-5 rounds of endocycles occur (Figure 1c, [15]). This analysis also highlights that in SR fruit the number of cells undergoing endoreduplication is lower than in FAN (Figure 1b), in agreement with the smaller size reached by SR fruit (Figure 1a). However, in peach the relationship between endoploidy and final fruit size must be considered in the light of a more recent evidence provided by Nilo-Poyanco et al. [55]. These authors, by using a proteomic approach on peach ripening fruit, identified a *P. persica* homologue of a GUANYLATE-BINDING PROTEIN1 (SlGBP1) (Prupe.4G053400, 70.6% identity), a tomato protein able to maintain endopolyploid cells in a non-proliferative state [56], which was accumulated with the progression of ripening. This result indicates that endopolyploid cells division would be inhibited in the last phase of peach fruit development [55] and is consistent with our cytofluorimetric analysis result on S4 FAN fruits (Figure 1c and Figure S1). Indeed, in S4 FAN fruit the proportion of clearly endoreduplicated cells (sum of 8C-32C populations) account for 39%, a much lower value than that observed in ripening tomato fruit, in which the number of endoreduplicated cells (sum of 8C-256C populations) was more than 85% of total pericarp cells [57]. On the basis of these observations, the endoreduplication contribution to the final peach fruit size seems to be modest in comparison to that observed in tomato, mainly due to the low number of endoreduplicated cells. In conclusion, the absolute number of cells seems to be the most important factor impacting peach final fruit size, as stated by Souza et al. [39] who evaluate the influence of cell number on final fruit size at about 60% (for cultivated variety). However, this is not the case for SR because the total number of fruit cell counted in this genotype is higher in comparison to FAN [24], but the higher cell number does not appear to be sufficient to rescue the reduced fruit size phenotype. This last observation indicates that other mechanisms (such as cell wall extensibility) might be responsible for SR defects in cell expansion and reduced final fruit size.

### 4.2. The Low Ploidy Level in SR Fruit Is Related to an Altered Expression of Genes Controlling the Endocycle Progression

To determine any possible causes for an altered endoreduplication in SR fruit cells, we investigated the expression of genes involved in the switching from a mitotic cell cycle to an endocycle. In this switch, a central role is played by kinase complexes, which in their minimal configuration consist of a CDK and a regulatory cyclin subunit [58]. CDK/cyclin activity is highly regulated at multiple levels. Control mechanisms include the regulated synthesis and destruction of cyclin subunits, which are thought to target the CDKs to the substrates, and the association of CDKs with inhibitory proteins and docking factors [59]. Moreover, CDK activity is negatively regulated by WEE1 kinases family phosphorylation [60]. An impaired expression of WEE1 in transgenic tomato plants resulted in a reduction of plant and fruit size [61]. The reduction of plant, fruit, and seed size originated from a reduction in cell size that was correlated with a decrease of the DNA ploidy levels, suggesting a role for WEE1 in endoreduplication progression. We observed that the major number of expression variations regard genes either with an inhibitory effect on cell cycle progression or involved in the switch to endoreduplication. In particular, the lack of a transient increase in WEE1 transcripts accumulation at the end of S1 in SR mesocarp cells in comparison to FAN (Figure 2E) may compromise the progression in the endocycle when, after pit hardening, the resumption of growth occurs. This view is supported by the fact that in SR the 8C cell population is comparable to that measured in FAN up to the end of the S2 stage (Figure 1c). Following the hypothesis that chromatin states are involved in the control of cell cycle progression, the alteration in transcription accumulation both of the cell cycle and the endocycle gene observed in SR fruit could be due to a different enrichment in histone marks between SR and FAN [62]. The persisting enrichment of H3K27me3 mark at WEE1 locus observed in SR fruit (Figure 5) suggests that the lack of the endocycle progression could be associated with a lower expression of WEE1 due to an inhibitory chromatin state [63].

### 4.3. Is the Altered Transcriptional Control of Cell Cycle and Endocycle Is Related to Uncorrected Fruit Patterning?

Recently, Tsukaya [64] reported that in leaves, the correlation between ploidy level and cell size is actively regulated by a genetic system that are linked to epidermal identity. In particular, he reported that an in situ single-cell-level analysis both of ploidy level and cell size demonstrated that there was not a clear correlation between cell size and ploidy level, in epidermal and subepidermal palisade cells of Arabidopsis leaves. Conversely, epidermal pavement cells size was positively correlated with endoreduplication. In tomato fruit, the cell ploidy level is higher in the pericarp and locular gel than the columella [57], indicating that in fruit the ploidy level is also linked to tissue identity. If ploidy level depends upon the tissue identity, we could hypothesize that the altered ploidy level of SR fruit mesocarp depends upon its alteration in tissue identity and to its resemblance to a lignified endocarp more than a mesocarp [24]. In SR fruit, mesocarp cells show higher accumulation of NST1 transcript, a NAC gene claimed to be a regulator of secondary wall formation and lignification, in endocarp cells [65]. For this role, NST1 has been indicated as an identity marker gene of lignified endocarp [66]. The higher level of NST1 transcripts in SR mesocarp cells is consistent with the higher level of lignin observed in this tissue in comparison to FAN [24]. In addition, in FAN, the lower transcript level of NST1 is associated with an enrichment in the H3K27me3 mark at its locus (Figure 6) suggesting that chromatin accessibility might change during tissue identity acquirement. Similarly, in SR mesocarp, the developmental stage marker genes are downregulated (Figure 3) and at their loci an enrichment in marks associated with a low chromatin accessibility is observed (Figure 6).

All these pieces of evidence suggest that the SR fruit phenotype, which is characterized by a higher cell density than FAN (Figure 1 and [24]) might be affected by a misregulation in cell identity: Indeed, SR mesocarp assumes an endocarp phenotype. SR endocarp cells are characterized by (1) a low endocycle number (the majority of cells are 4C) associated with a lack of transient accumulation of WEE1, and (2) a high lignin content due to an extension of NST1 transcripts accumulation during the S3 developmental stage. The cell wall lignification of SR mesocarp cells negatively impacts the cell capability to undergo expansion and, consequently, to reach a regular final fruit size (Figure 1a,b). The impaired expression of genes controlling cell cycle and endocycle, as well as those claimed to play a role in fruit cell tissue identity, are under the control of molecular mechanisms acting at chromatin level. Our results indicate chromatin marks as potential actors in the regulation of mesocarp endoreduplication events: Future research based on genome-wide chromatin analysis will clarify whether epigenetic signatures control mesocarp differentiation and growth dynamics in peach.

## Data Availability

Data is available in the manucscript and Supplementary Materials.

## Supplementary Materials

The following are available online at https://www.mdpi.com/article/10.3390/genes12040482/s1, Figure S1: Ploidy distribution in mesocarp cells of FAN (up) and SR (down) fruits. Figure S2: Expression pattern of peach ortholog of NAC (Prupe.4G186800) gene throughout the whole fruit development in the mesocarp of cv Fantasia and Slow Ripening fruit; Table S1: Primers RT-PCR and ChIP analyses. Table S2: Significant differences (*t*-test values) in terms of mesocarp cell ploidy between FAN and SR.

## References

[1] Yao J.-L., Xu J., Tomes S., Cui W., Luo Z., Deng C., Ireland H.S., Schaffer R.J., Gleave A.P. (2018). Ectopic Expression of the PISTILLATA Homologous MdPI Inhibits Fruit Tissue Growth and Changes Fruit Shape in Apple. Plant Direct.

[2] Harada T., Kurahashi W., Yanai M., Wakasa Y., Satoh T. (2005). Involvement of Cell Proliferation and Cell Enlargement in Increasing the Fruit Size of Malus Species. Sci. Hortic..

[3] Olmstead J.W., Iezzoni A.F., Whiting M.D. (2007). Genotypic Differences in Sweet Cherry Fruit Size Are Primarily a Function of Cell Number. J. Am. Soc. Hortic. Sci..

[4] Yamaguchi M., Haji T., Miyake M., Yaegaki H. (2002). Varietal Differences in Cell Division and Enlargement Periods during Peach (*Prunus persica* Batsch) Fruit Development. J. Jpn. Soc. Hortic. Sci..

[5] Scorza R., May L.G., Purnell B., Upchurch B. (1991). Differences in Number and Area of Mesocarp Cells between Small- and Large-Fruited Peach Cultivars. J. Am. Soc. Hortic. Sci. Jashs.

[6] Masia A., Zanchin A., Rascio N., Ramina A. (1992). Some Biochemical and Ultrastructural Aspects of Peach Fruit Development. J. Am. Soc. Hortic. Sci. Jashs.

[7] Bonghi C., Ferrarese L., Ruperti B., Tonutti P., Ramina A. (1998). Endo-β-1,4-Glucanases Are Involved in Peach Fruit Growth and Ripening, and Regulated by Ethylene. Physiol. Plant..

[8] Rodriguez C.E., Bustamante C.A., Budde C.O., Muller G.L., Drincovich M.F., Lara M.V. (2019). Peach Fruit Development: A Comparative Proteomic Study Between Endocarp and Mesocarp at Very Early Stages Underpins the Main Differential Biochemical Processes Between These Tissues. Front. Plant Sci..

[9] Zanchin A., Bonghi C., Casadoro G., Ramina A., Rascio N. (1994). Cell Enlargement and Cell Separation During Peach Fruit Development. Int. J. Plant Sci..

[10] Ohmiya A. (2000). Effects of Auxin on Growth and Ripening of Mesocarp Discs of Peach Fruit. Sci. Hortic..

[11] Shi M., Hu X., Wei Y., Hou X., Yuan X., Liu J., Liu Y. (2017). Genome-Wide Profiling of Small RNAs and Degradome Revealed Conserved Regulations of MiRNAs on Auxin-Responsive Genes during Fruit Enlargement in Peaches. Int. J. Mol. Sci..

[12] Bertin N. (2005). Analysis of the Tomato Fruit Growth Response to Temperature and Plant Fruit Load in Relation to Cell Division, Cell Expansion and DNA Endoreduplication. Ann. Bot..

[13] Rewers M., Sadowski J., Sliwinska E. (2009). Endoreduplication in Cucumber (*Cucumis sativus*) Seeds during Development, after Processing and Storage, and during Germination. Ann. Appl. Biol..

[14] Chevalier C., Nafati M., Mathieu-Rivet E., Bourdon M., Frangne N., Cheniclet C., Renaudin J.-P., Gevaudant F., Hernould M. (2011). Elucidating the Functional Role of Endoreduplication in Tomato Fruit Development. Ann. Bot..

[15] Bourdon M., Frangne N., Mathieu-Rivet E., Nafati M., Cheniclet C., Renaudin J.P., Chevalier C. (2010). Endoreduplication and growth of fleshy fruits. Progress in Botany.

[16] Brecht J.K., Kader A.A. (1984). Ethylene Production by Fruit of Some Slow-Ripening Nectarine Genotypes. J. Am. Soc. Hortic. Sci..

[17] Brecht J.K., Kader A.A., Ramming D.W. (1984). Description and Postharvest Physiology of Some Slow-Ripening Nectarine Genotypes. J. Am. Soc. Hortic. Sci..

[18] Ramming D.W. (1991). Genetic Control of a Slow-Ripening Fruit Trait in Nectarine. Can. J. Plant Sci..

[19] Tataranni G., Spada A., Pozzi C., Bassi D. (2010). AFLP-Based Bulk Segregant Analysis for Tagging the Slow-Ripening Trait in Peach [*Prunus persica* (L.) Batsch]. J. Hortic. Sci. Biotechnol..

[20] Giné-Bordonaba J., Eduardo I., Arús P., Cantín C.M. (2020). Biochemical and Genetic Implications of the Slow Ripening Phenotype in Peach Fruit. Sci. Hortic..

[21] Eduardo I., Picañol R., Rojas E., Batlle I., Howad W., Aranzana M.J., Arús P. (2015). Mapping of a Major Gene for the Slow Ripening Character in Peach: Co-Location with the Maturity Date Gene and Development of a Candidate Gene-Based Diagnostic Marker for Its Selection. Euphytica.

[22] Meneses C., Ulloa-Zepeda L., Cifuentes-Esquivel A., Infante R., Cantin C.M., Batlle I., Arús P., Eduardo I. (2016). A Codominant Diagnostic Marker for the Slow Ripening Trait in Peach. Mol. Breed..

[23] Nuñez-Lillo G., Cifuentes-Esquivel A., Troggio M., Micheletti D., Infante R., Campos-Vargas R., Orellana A., Blanco-Herrera F., Meneses C. (2015). Identification of Candidate Genes Associated with Mealiness and Maturity Date in Peach [*Prunus persica* (L.) Batsch] Using QTL Analysis and Deep Sequencing. Tree Genet. Genomes.

[24] Botton A., Rasori A., Ziliotto F., Moing A., Maucourt M., Bernillon S., Deborde C., Petterle A., Varotto S., Bonghi C. (2016). The Peach HECATE3-like Gene FLESHY Plays a Double Role during Fruit Development. Plant Mol. Biol..

[25] Gu C., Zhou Y.-H., Shu W.-S., Cheng H.-Y., Wang L., Han Y.-P., Zhang Y.-Y., Yu M.-L., Joldersma D., Zhang S.-L. (2018). RNA-Seq Analysis Unveils Gene Regulation of Fruit Size Cooperatively Determined by Velocity and Duration of Fruit Swelling in Peach. Physiol. Plant..

[26] Zhou H., Lin-Wang K., Wang H., Gu C., Dare A.P., Espley R.V., He H., Allan A.C., Han Y. (2015). Molecular Genetics of Blood-Fleshed Peach Reveals Activation of Anthocyanin Biosynthesis by NAC Transcription Factors. Plant J. Cell Mol. Biol..

[27] Dirlewanger E., Moing A., Rothan C., Svanella L., Pronier V., Guye A., Plomion C., Monet R. (1999). Mapping QTLs Controlling Fruit Quality in Peach (*Prunus persica* (L.) Batsch). Theor. Appl. Genet..

[28] Quilot B., Wu B.H., Kervella J., Genard M., Foulongne M., Moreau K. (2004). QTL Analysis of Quality Traits in an Advanced Backcross between *Prunus persica* Cultivars and the Wild Relative Species *P. davidiana*. TAG Theor. Appl. Genet. Theor. Angew. Genet..

[29] Martinez-Garcia P.J., Parfitt D.E., Bostock R.M., Fresnedo-Ramirez J., Vazquez-Lobo A., Ogundiwin E.A., Gradziel T.M., Crisosto C.H. (2013). Application of Genomic and Quantitative Genetic Tools to Identify Candidate Resistance Genes for Brown Rot Resistance in Peach. PLoS ONE.

[30] Zeballos J.L., Abidi W., Giménez R., Monforte A.J., Moreno M.Á., Gogorcena Y. (2016). Mapping QTLs Associated with Fruit Quality Traits in Peach [*Prunus persica* (L.) Batsch] Using SNP Maps. Tree Genet. Genomes.

[31] Marshall W.F., Young K.D., Swaffer M., Wood E., Nurse P., Kimura A., Frankel J., Wallingford J., Walbot V., Qu X. (2012). What Determines Cell Size?. BMC Biol..

[32] Gu Q., Ferrandiz C., Yanofsky M.F., Martienssen R. (1998). The FRUITFULL MADS-Box Gene Mediates Cell Differentiation during Arabidopsis Fruit Development. Dev. Camb. Engl..

[33] Kim Y.-S., Kim S.-G., Park J.-E., Park H.-Y., Lim M.-H., Chua N.-H., Park C.-M. (2006). A Membrane-Bound NAC Transcription Factor Regulates Cell Division in Arabidopsis. Plant Cell.

[34] Li C., Potuschak T., Colon-Carmona A., Gutierrez R.A., Doerner P. (2005). Arabidopsis TCP20 Links Regulation of Growth and Cell Division Control Pathways. Proc. Natl. Acad. Sci. USA.

[35] Fukazawa J., Sakai T., Ishida S., Yamaguchi I., Kamiya Y., Takahashi Y. (2000). Repression of Shoot Growth, a BZIP Transcriptional Activator, Regulates Cell Elongation by Controlling the Level of Gibberellins. Plant Cell.

[36] Nibau C., Gibbs D.J., Bunting K.A., Moody L.A., Smiles E.J., Tubby J.A., Bradshaw S.J., Coates J.C. (2011). ARABIDILLO Proteins Have a Novel and Conserved Domain Structure Important for the Regulation of Their Stability. Plant Mol. Biol..

[37] Weber C.M., Henikoff S. (2014). Histone Variants: Dynamic Punctuation in Transcription. Genes Dev..

[38] Catarino R.R., Stark A. (2018). Assessing Sufficiency and Necessity of Enhancer Activities for Gene Expression and the Mechanisms of Transcription Activation. Genes Dev..

[39] Souza F., Alves E., Pio R., Castro E., Reighard G., Freire A.I., Mayer N.A., Pimentel R. (2019). Influence of Temperature on the Development of Peach Fruit in a Subtropical Climate Region. Agronomy.

[40] Ziliotto F., Corso M., Rizzini F.M., Rasori A., Botton A., Bonghi C. (2012). Grape Berry Ripening Delay Induced by a Pre-Veraison NAA Treatment Is Paralleled by a Shift in the Expression Pattern of Auxin- and Ethylene- Related Genes. BMC Plant Biol..

[41] Giulia E., Alessandro B., Mariano D., Andrea B., Benedetto R., Angelo R. (2013). Early Induction of Apple Fruitlet Abscission Is Characterized by an Increase of Both Isoprene Emission and Abscisic Acid Content. Plant Physiol..

[42] Simon P. (2003). Q-Gene: Processing Quantitative Real-Time RT-PCR Data. Bioinform. Oxf. Engl..

[43] Pfaffl M.W. (2001). A New Mathematical Model for Relative Quantification in Real-Time RT-PCR. Nucleic Acids Res..

[44] Tong Z., Gao Z., Wang F., Zhou J., Zhang Z. (2009). Selection of Reliable Reference Genes for Gene Expression Studies in Peach Using Real-Time PCR. BMC Mol. Biol..

[45] Bonghi C., Trainotti L., Botton A., Tadiello A., Rasori A., Ziliotto F., Zaffalon V., Casadoro G., Ramina A. (2011). A Microarray Approach to Identify Genes Involved in Seed-Pericarp Cross- Talk and Development in Peach. BMC Plant Biol..

[46] Pfaffl M.W., Tichopad A., Prgomet C., Neuvians T.P. (2004). Determination of Stable Housekeeping Genes, Differentially Regulated Target Genes and Sample Integrity: BestKeeper--Excel-Based Tool Using Pair-Wise Correlations. Biotechnol. Lett..

[47] Rossi V., Locatelli S., Varotto S., Donn G., Pirona R., Henderson D.A., Hartings H., Motto M. (2007). Maize Histone Deacetylase Hda101 Is Involved in Plant Development, Gene Transcription, and Sequence-Specific Modulation of Histone Modification of Genes and Repeats. Plant Cell.

[48] Tonutti P., Bonghi C., Ruperti B., Tornielli G.B., Ramina A. (1997). Ethylene Evolution and 1-Aminocyclopropane-1-Carboxylate Oxidase Gene Expression during Early Development and Ripening of Peach Fruit. J. Am. Soc. Hortic. Sci. Jashs.

[49] Malladi A., Hirst P.M. (2010). Increase in Fruit Size of a Spontaneous Mutant of “Gala” Apple (Malus x Domestica Borkh.) Is Facilitated by Altered Cell Production and Enhanced Cell Size. J. Exp. Bot..

[50] Qi X., Liu C., Song L., Li Y., Li M. (2017). PaCYP78A9, a Cytochrome P450, Regulates Fruit Size in Sweet Cherry (*Prunus avium* L.). Front. Plant Sci..

[51] Dardick C.D., Callahan A.M., Chiozzotto R., Schaffer R.J., Piagnani M.C., Scorza R. (2010). Stone Formation in Peach Fruit Exhibits Spatial Coordination of the Lignin and Flavonoid Pathways and Similarity to Arabidopsis Dehiscence. BMC Biol..

[52] Farinati S., Rasori A., Varotto S., Bonghi C. (2017). Rosaceae Fruit Development, Ripening and Post-Harvest: An Epigenetic Perspective. Front. Plant Sci..

[53] Forestan C., Varotto S., Bonghi C. (2019). Personal communication.

[54] Chevalier C., Bourdon M., Pirrello J., Cheniclet C., Gevaudant F., Frangne N. (2014). Endoreduplication and Fruit Growth in Tomato: Evidence in Favour of the Karyoplasmic Ratio Theory. J. Exp. Bot..

[55] Nilo-Poyanco R., Moraga C., Benedetto G., Orellana A., Almeida A.M. (2021). Shotgun Proteomics of Peach Fruit Reveals Major Metabolic Pathways Associated to Ripening. BMC Genom..

[56] Musseau C., Jorly J., Gadin S., Sorensen I., Deborde C., Bernillon S., Mauxion J.-P., Atienza I., Moing A., Lemaire-Chamley M. (2020). The Tomato Guanylate-Binding Protein SlGBP1 Enables Fruit Tissue Differentiation by Maintaining Endopolyploid Cells in a Non-Proliferative State. Plant Cell.

[57] Cheniclet C., Rong W.Y., Causse M., Frangne N., Bolling L., Carde J.-P., Renaudin J.-P. (2005). Cell Expansion and Endoreduplication Show a Large Genetic Variability in Pericarp and Contribute Strongly to Tomato Fruit Growth. Plant Physiol..

[58] Verkest A., Weinl C., Inze D., De Veylder L., Schnittger A. (2005). Switching the Cell Cycle. Kip-Related Proteins in Plant Cell Cycle Control. Plant Physiol..

[59] Lees E. (1995). Cyclin Dependent Kinase Regulation. Curr. Opin. Cell Biol..

[60] Berry L.D., Gould K.L. (1996). Novel Alleles of Cdc13 and Cdc2 Isolated as Suppressors of Mitotic Catastrophe in Schizosaccharomyces Pombe. Mol. Gen. Genet. MGG.

[61] Gonzalez N., Gevaudant F., Hernould M., Chevalier C., Mouras A. (2007). The Cell Cycle-Associated Protein Kinase WEE1 Regulates Cell Size in Relation to Endoreduplication in Developing Tomato Fruit. Plant J. Cell Mol. Biol..

[62] Desvoyes B., Fernandez-Marcos M., Sequeira-Mendes J., Otero S., Vergara Z., Gutierrez C. (2014). Looking at Plant Cell Cycle from the Chromatin Window. Front. Plant Sci..

[63] Shu H., Wildhaber T., Siretskiy A., Gruissem W., Hennig L. (2012). Distinct Modes of DNA Accessibility in Plant Chromatin. Nat. Commun..

[64] Tsukaya H. (2019). Re-Examination of the Role of Endoreduplication on Cell-Size Control in Leaves. J. Plant Res..

[65] Mitsuda N., Ohme-Takagi M. (2008). NAC Transcription Factors NST1 and NST3 Regulate Pod Shattering in a Partially Redundant Manner by Promoting Secondary Wall Formation after the Establishment of Tissue Identity. Plant J. Cell Mol. Biol..

[66] Dardick C., Callahan A.M. (2014). Evolution of the Fruit Endocarp: Molecular Mechanisms Underlying Adaptations in Seed Protection and Dispersal Strategies. Front. Plant Sci..

