# Supplementary material for: Regulation of Fruit Growth in a Peach Slow Ripening Phenotype
Source: Genes (Basel). 2021 Mar 26;12(4):482. doi: 10.3390/genes12040482 (PMC8066772; doi:10.3390/genes12040482)

Figure S1

Expression pattern of peach ortholog of NAC (Prupe.4G186800) gene throughout the whole fruit development in the mesocarp of cv Fantasia and Slow Ripening fruit

Real time qPCR expression analysis of peach ortholog of NAC (Prupe.4G186800) gene throughout fruit development in the mesocarp of cv FAN (square, dashed line) and SR (circle, continous line) (for details see Methods). Developmental stages (S1, S2, S3, and S4) are indicated at the top of each chart, taking FAN as a reference. Highly significant differences as determined by Student's T test are indicated with two ($P \leq 0.01$) asterisks, respectively. MNE: Mean normalized expression, DAFB: Days After Full Bloom. Bars represent standard deviation (n=3).

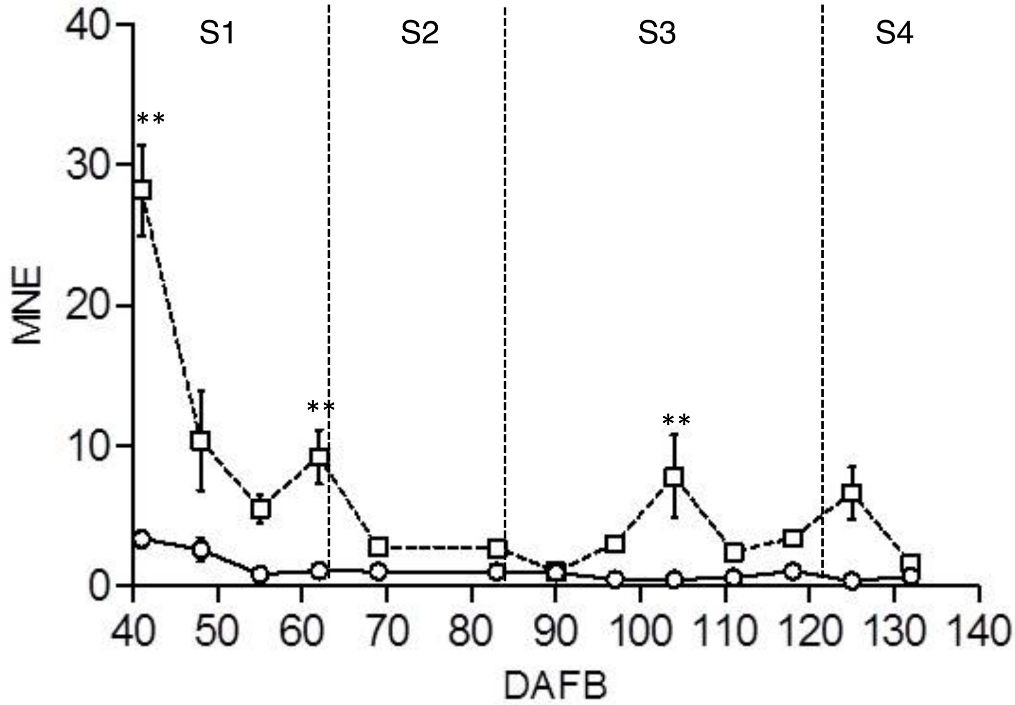

Figure S2. Ploidy distribution in mesocarp cells of FAN (up) and SR (down) fruits.

The frequency data of each C-value class derive from 15 fruits at different developmental stages. Developmental stages (S1, S2, S3, and S4) are indicated at the top of each chart, taking FAN as a reference. DAFB, Days After Full Bloom. PH, pit hardening.

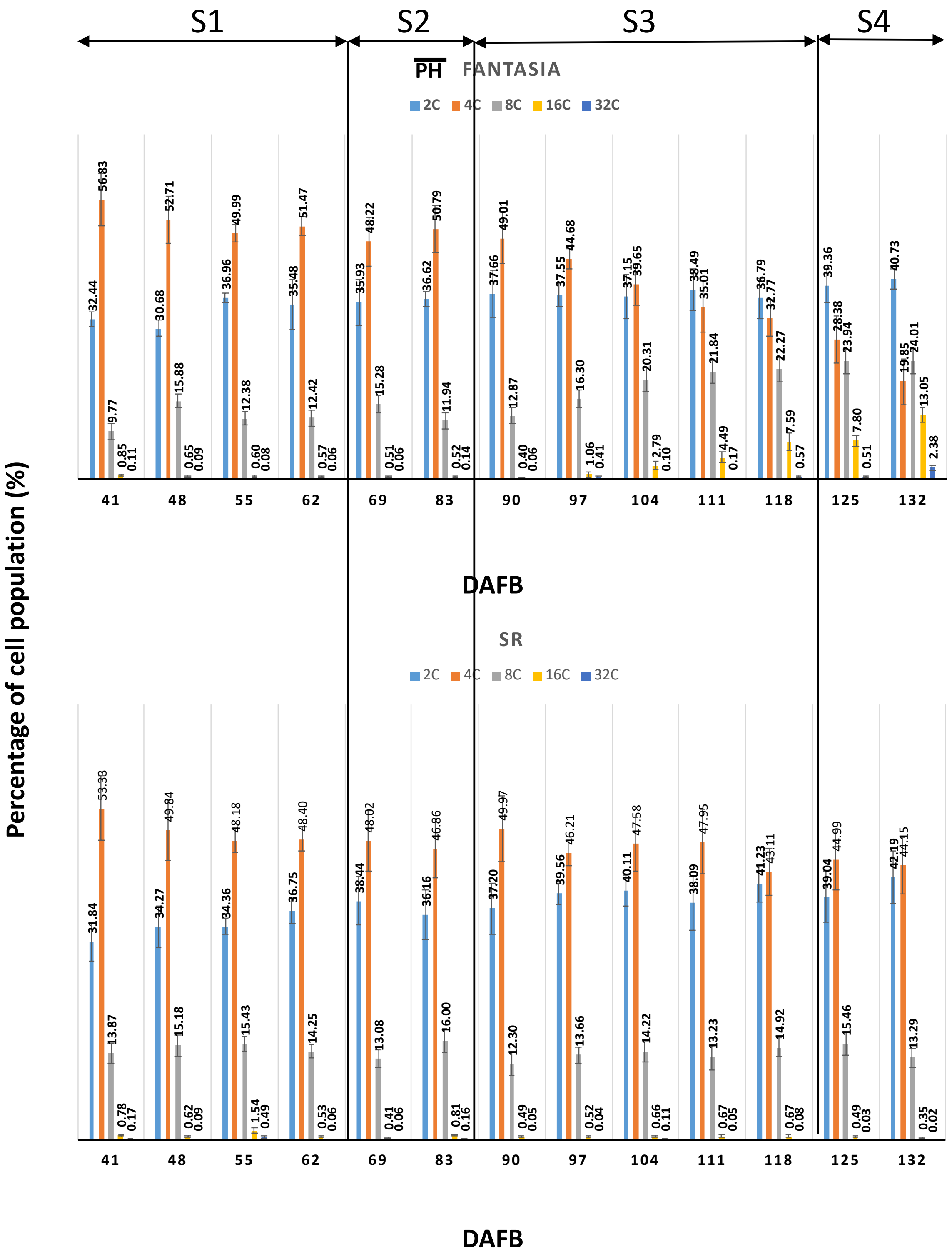

Supplement: Supplementary file 1 [file genes-12-00482-s001.zip › Figure S1_S2.pdf]
