# Supplementary material for: Regulation of Fruit Growth in a Peach Slow Ripening Phenotype
Source: Genes (Basel). 2021 Mar 26;12(4):482. doi: 10.3390/genes12040482 (PMC8066772; doi:10.3390/genes12040482)
Supplement: Supplementary file 1 [file genes-12-00482-s001.zip › Table S1.pdf]

Table S1: Primers RT-PCR and ChIP analyses

Primer sequences used for expression and ChIP analyses. Oligonucleotides were designed, for expression RT-PCR, on primary transcript corresponding to target gene sequences, including the housekeeping genes, and close to annotated genomic TSS (from http://phytozome.jgi.doe.gov) for single loci for the ChIP investigation. All the sequences are reported in a 5'-3' orientation, grouped according to main categories.

qPCR analisyses			ChIP analyses	
***P. persica* ID**	**TAIR**	**Primer sequence 5'>3'**	**Primer sequence 5'>3'**	**ChIP Primers coordinates compared to TSS**
**Target genes**				
**Prupe.3G075200_CYCA2/3 related**	CYCA2;3	Fw: AGGCTGTGCGAACAAATCCA<br>Rev: CAGCAAGCATTGGTCACATCC	Fw: AACATCTTGAAATGAGCCCACTTCGACATG<br>Rev: GTCCCAAATAATCTTTCTGAGCAAACTCCC	**TSS** +612bp (Fw) +779bp (Rev) (5'UTR)
**Prupe.1G428100_CYCA related**	CYCA3;1	Fw: CCAAAGCCAAGGCGAAGAAA<br>Rev: AATCATAAATGTCACGAGCATAAGG	Fw: GAAGCAATTCAAAGCAGGGAACCAAAATGG<br>Rev: CCCTAGCGAATCTGTGAGTGTCGAGG	-56bp (Fw) **TSS** +137 (Rev) (5'UTR)
**Prupe.2G084600_CDKA-type**	CDK2	Fw: CGTGGTTTACAAGGCTCGTG<br>Rev: GCCTGACAATGTTGCCATGTT	Fw: GCTGGATGTCACCGCTGGAGCTG<br>Rev: CTGCTACACAACAGTACCTGACCGC	-47bp (Fw) **TSS** +185 bp (Rev) (5'UTR)
**Prupe.6G299900_CDKB-type**	CDKB1	Fw: CCTCGACACCGATCTCAAGAA<br>Rev: CCCTTGCACAGTTGGTAGAGGA	Fw: GACATTGATGTACCATTATTACGTTGC<br>Rev: CGTGTCCCGCGCTTGCCTTCACG	-104 bp (Fw) **TSS** +200bp (Rev) (5'UTR)
**Prupe.1G006300_CKI**	KRP3	Fw: CATCGGCTTCACCTGTCACC<br>Rev: GCCTCGAAGTCGCCCTAGAA	Fw: GGCTCTAGAGGCTGCCACTTACTAC<br>Rev: CCTCATGTACTTGCCCATTTCTTGCTC	-9bp **TSS** +16bp (Fw) +242bp (Rev) (5'UTR and coding sequence)
**Prupe.8G256300_WEE1-like protein**	WEE1	Fw: GCTTGGTGACTTTGGATGTGC<br>Rev: TGGCAACTCCCAAGGAGAAGA	Fw: CTGCTCCTGATGTGATTTATGCCC<br>Rev: GACAATTGGTGGCTTTCACCTAGC	-0 (Fw) **TSS** +221 bp (Rev) (coding sequence)
**Prupe.5G131900_NST1**	NST1	Fw: ACGGCCAGTCTCAAGTTCCTCCG<br>Rev: CGAATCACATCAAGATCAATCCTCTC	Fw: GTGGTTGGCTCTTCTTCAAGGGATGG<br>Rev: CGGTGAGAGAATGGAATTGAAGGAGCTAG	-86bp (Fw) **TSS** +108 bp (Rev) (coding sequence)
**Prupe.5G117500_CABP-like**	CABP-like	Fw: CCCGCGACCGCCATACTCTTAC<br>Rev: TCAACGGAACGGACTCGCTGTG	Fw: CACCACCAACCGCTCATCTTCTCC<br>Rev: CGTTGGTTTCCTTCTCGGGTAGATCGG	-15 bp **TSS** + 9bp (Fw) +122bp (Rev) (coding sequence)
**Prupe.8G232200_Aux/IAA**	AUX_IAA	Fw: CCAAGACCAACAAGGACAACA<br>Rev: CATGCTCACCTTCACCA	Fw: TGTCCAGAGAGTGCAGTGTCCACG<br>Rev: GGGCTGCGTCGGTATTAATATGCG	-24 bp (Fw) **TSS** +168 bp (Rev) (5'UTR)
**Prupe.4G186800_NAC**	NAC	Fw: CGAACCTTCACGCAAAAATGGCAGCT<br>Rev: CGGTCGTCAATATCCGGGAGC		
**Housekeeping genes:**				
**Prupe.1G364800_TUB**	TUB	Fw: CCGAGAATTGTGACTGCCTTCAAG<br>Rev: AGCATCATCCTGTCTGGGTATTCC		
**Prupe.4G204900_UBQ10**	UBQ10	Fw: AAGGCTAAGATCCAAGACAAAGAG<br>Rev: CCACGAAGACGAAGCACTAAG		
**Prupe.8G137600_PPN1**	PPN1	Fw: CCAGGAGAATCGGTGAGCAGAAAA<br>Rev: TCGAGGGTGGAGGACTTGAGAATG		
