# Supplementary material for: Regulation of Fruit Growth in a Peach Slow Ripening Phenotype
Source: Genes (Basel). 2021 Mar 26;12(4):482. doi: 10.3390/genes12040482 (PMC8066772; doi:10.3390/genes12040482)
Supplement: Supplementary file 1 [file genes-12-00482-s001.zip › Table S2.pdf]

Table S2. T-test (two-tailed distribution, heteroscedastic) was used to calculate statistical significance of difference in the composition of cell populations, in terms of DNA content (* : 0.01; ** : 0.05; *** : 0.001).

Fruit development stage	sampling date (DAFB)	fruit	p-value	significance
S1	41	M1	5.75E-01	
		M2	9.51E-02	
		M3	4.89E-02	
		M4	6.79E-01	
		M5	3.19E-01	
	48	M1	4.86E-03	**
		M2	1.31E-01	
		M3	6.80E-01	
		M4	6.83E-01	
		M5	8.23E-01	
	55	M1	2.19E-01	
		M2	4.03E-01	
		M3	1.16E-01	
		M4	2.37E-02	
		M5	1.49E-01	
	62	M1	5.93E-01	
		M2	2.19E-01	
		M3	2.39E-01	
		M4	6.44E-01	
		M5	9.55E-01	
S2	69	M1	1.78E-01	
		M2	9.14E-01	
		M3	1.57E-01	
		M4	1.87E-01	
		M5	8.40E-01	
	83	M1	8.53E-01	
		M2	1.04E-01	
		M3	6.64E-02	
		M4	7.90E-02	
		M5	6.62E-01	
S3	90	M1	8.46E-01	
		M2	7.23E-01	
		M3	7.63E-01	
		M4	5.22E-01	
		M5	8.01E-01	
	97	M1	3.42E-01	
		M2	6.52E-01	
		M3	2.50E-01	
		M4	3.31E-01	
		M5	3.43E-01	
	104	M1	3.08E-01	
		M2	2.15E-01	
		M3	5.98E-01	
		M4	2.74E-01	
		M5	2.30E-01	
	111	M1	8.89E-01	
		M2	2.45E-03	**
		M3	2.23E-04	***
		M4	4.46E-03	**
		M5	4.35E-02	
	118	M1	1.92E-01	
		M2	4.11E-02	
		M3	2.39E-03	**
		M4	2.10E-03	**
		M5	9.44E-03	*
S4	125	M1	8.80E-01	
		M2	7.12E-05	***
		M3	1.25E-05	***
		M4	3.42E-05	***
		M5	3.13E-03	**
	132	M1	5.51E-01	
		M2	2.15E-08	***
		M3	1.60E-06	***
		M4	2.41E-07	***
		M5	1.05E-03	**
